# Trends in the Design of New Isobaric Labeling Reagents for Quantitative Proteomics

**DOI:** 10.3390/molecules24040701

**Published:** 2019-02-15

**Authors:** Remigiusz Bąchor, Mateusz Waliczek, Piotr Stefanowicz, Zbigniew Szewczuk

**Affiliations:** Faculty of Chemistry, University of Wrocław, F. Joliot-Curie 14, 50-383 Wrocław, Poland; mateusz.waliczek@chem.uni.wroc.pl (M.W.); piotr.stefanowicz@chem.uni.wroc.pl (P.S.)

**Keywords:** isobaric labeling reagents, quantitative proteomics, ESI-MS, ionization enhancers, quaternary ammonium salts

## Abstract

Modern mass spectrometry is one of the most frequently used methods of quantitative proteomics, enabling determination of the amount of peptides in a sample. Although mass spectrometry is not inherently a quantitative method due to differences in the ionization efficiency of various analytes, the application of isotope-coded labeling allows relative quantification of proteins and proteins. Over the past decade, a new method for derivatization of tryptic peptides using isobaric labels has been proposed. The labels consist of reporter and balanced groups. They have the same molecular weights and chemical properties, but differ in the distribution of stable heavy isotopes. These tags are designed in such a way that during high energy collision induced dissociation (CID) by tandem mass spectrometry, the isobaric tag is fragmented in the specific linker region, yielding reporter ions with different masses. The mass shifts among the reporter groups are compensated by the balancing groups so that the overall mass is the same for all forms of the reagent. Samples of peptides are labeled with the isobaric mass tags in parallel and combined for analysis. Quantification of individual peptides is achieved by comparing the intensity of reporter ions in the tandem mass (MS/MS) spectra. Isobaric markers have found a wide range of potential applications in proteomics. However, the currently available isobaric labeling reagents have some drawbacks, such as high cost of production, insufficient selectivity of the derivatization, and relatively limited enhancement of sensitivity of the analysis. Therefore, efforts have been devoted to the development of new isobaric markers with increased usability. The search for new isobaric markers is focused on developing a more selective method of introducing a tag into a peptide molecule, increasing the multiplexicity of markers, lowering the cost of synthesis, and increasing the sensitivity of measurement by using ionization tags containing quaternary ammonium salts. Here, the trends in the design of new isobaric labeling reagents for quantitative proteomics isobaric derivatization strategies in proteomics are reviewed, with a particular emphasis on isobaric ionization tags. The presented review focused on different types of isobaric reagents used in quantitative proteomics, their chemistry, and advantages offer by their application.

## 1. Introduction

Mass spectrometry (MS) has become a powerful tool for the analysis of complex protein mixtures. This method combined with various separation techniques offers extremely high resolution and sensitivity; however, obtaining quantitative results is often problematic. Nowadays the problem of quantification is solved in two ways: using label free methods based on spectral count or chromatographic peak area/height, or by methods using stable isotopes. The literature data suggest that the label-free method is more efficient in terms of number of identified and quantified proteins; however, isotopic methods are characterized by better reproducibility and precision [1]. Another advantage of the methods based on isotopic labeling is multiplexing, allowing parallel analysis of several samples, which saves analysis time and increases the overall reliability of the method.

The incorporation of isotopic label may be performed on various stages of analytical procedure. Currently used methods allow the labeling of intact proteins or the peptides obtained by the enzymatic hydrolysis of the analyzed sample. The labeling of whole proteins is often based on biological systems including cell cultures (SILAC—stable isotope labeling by amino acids in cell culture) [2], or whole organisms (SILAM—stable isotope labeling in mammals [3], HILEP—hydroponic isotope labeling of entire plants [4]). The recombinant proteins or constructs composed of many sequences corresponding to peptides present in the analyzed sample (QconCAT—quantitative concatenation) may be used as isotopically labeled standards [5]. The labeling of whole proteins has many advantages, since standard and sample may be combined before analytical separation, e.g., by electrophoresis, and this procedure is not compromised for the incomplete tryptic hydrolysis.

The most frequently used method uses chemical modification of peptides obtained by the enzymatic hydrolysis of protein sample. The specially designed reagents target amino, sulfhydryl and carboxyl groups of peptides. These labeling reagents incorporate stable heavy isotopes like ^13^C, ^15^N, ^18^O and ^2^H. The incorporation of isotopic label should result in formation of two isotopologues of peptide, which have the same chemical and chromatographic properties, but differ in molecular masses and can be distinguished in the MS spectrum, allowing direct comparison of the abundance of peptide in labeled and unlabeled sample. It is worth noting that the deuterium isotope is easily available and relatively inexpensive, but may affect a retention time of isotopologues during chromatographic separation of labeled peptides [6].

The isotopic dilution method is efficient in the analysis of samples of moderate complexity. However, the proteomic samples often contain thousands of components. In such situations, the presence of compounds with similar molecular masses and retention times may cause severe quantification errors resulting from the overlapping of the matrix component peaks and peaks of analyte or isotopically labeled standards. Moreover, the system based on the mass differences caused by isotopic substitution is limited to a binary (2-plex), ternary (3-plex) or quaternary (4-plex) set of tags. Therefore, the comparison of samples corresponding to multiple states cannot be achieved in one experiment [7]. The problem of multiplicity in peptide analysis was solved by designing the sets of isobaric reagents with different distribution of heavy isotopes. The products obtained by chemical modification of peptides with these labels are isobaric, with the same chemical and physical properties, and therefore are chromatographically indistinguishable. The relative abundancies of two isotopic variants of peptide is revealed in MS/MS experiment, when fragmentation produces reporter ions with different molecular masses. At the same time, the peptide fragment ions are used for identification of peptide sequences characteristic for the analyzed protein. The structure of isobaric reagents is in general composed of three groups. First of all, they contain a reporter group, a moiety which is easily released from the labeled peptide during collision induced dissociation (CID). The reporter groups may have different molecular masses because of isotopic substitution. This difference allows distinguishing and quantification of peptides from various samples. Another component of the isobaric reagent is a balance group. The balance group is also isotopically substituted assuring that the total mass of the reporter ion and the balance group is the same in all versions of the reagent. The isobaric reagents contain also a reactive group —the moiety responsible for selective reaction of reagent with selected functional groups in a peptide. The most common reactive group is the succinimidyl ester, which reacts with moderate selectivity with amino groups in peptides forming stable amide bond [8]. The active esters react also with phenolic group of tyrosine, imidazole in histidine side chain as well as SH group of cysteine. There are also reports on applying other reactive groups like pentafluorophenyl esters [9], dimetoxytriazyne esters [10], groups selective for thiol moiety, e.g., iodoacetamide [11] and groups targeting carbonyl moiety in carbonylated proteins, e.g., substituted hydrazines [12] and hydroxylamines [13].

The first example of isobaric tag, *Tandem Mass Tag* (TMT, duplex), was demonstrated by Thomson [14] who has shown that in this strategy, a very high signal-to-noise ratio is achieved compared to MS1 mode. The structure of TMT reagent was a relatively complex and involved deuterium substitution, which may lead to the chromatographic separation of labeled peptides and quantification errors. Another example of isobaric tag, isobaric tags for relative and absolute quantification (iTRAQ), was demonstrated by Ross et al. who tested this reagent for comparison of protein expression in isogenic yeast strains [15]. The isobaric iTRAQ in version developed by Ross was available as a tetraplex. Over the past decade, chemical labeling with isobaric tandem mass tags, such as iTRAQ and TMT reagents, has been employed in a wide range of different clinically orientated serum and plasma proteomics studies [16].

Currently, labeling with isobaric tags is a routine method in quantitative proteomics studies, including comparative proteomics and absolute protein quantification. However, there are some limitations of this group of reagents.

The recent papers demonstrate many examples of the modified isobaric tags. The main directions in development of a new classes of isobaric labeling reagents for quantitative proteomics include:

- Increase in sensitivity of detection of the labeled peptides. This goal may be accomplished by incorporating the permanent electric charge to the labeled molecule, which facilitates electrospray ionization (ESI).
- Increase in the multiplexing capacity of isobaric markers. This modification allows to compare bigger number of proteomic samples in a single experiment.
- Modification of reactive group in isobaric tags in order to increase the selectivity and yield of derivatization.
- Simplification of the structure of isobaric tag in order to reduce prices of this group of reagents.

These trends in a development of isobaric tags will be discussed herein.

The mass spectrometry-based quantitative proteomics can be applied as an absolute or relative method. The first of them allows the quantification of changes in protein expression, whereas the second one is commonly use to establish the protein downregulation in a relation to the control sample. Therefore, in the relative type of analysis, the introduction of a chemically equivalent differential isotopic mass tag is required for comparative quantitation of proteins in different samples. The role of the applied tag consists of the protein or peptide mass change without peptide or protein molecules; this may be achieved by metabolic or in vivo labeling, involving the incorporation of stable isotopes during protein biosynthesis, enzymatic reaction or chemical modification [17]. Among all the developed and applied methods for quantitative proteomic analysis, chemical labeling with isobaric tags is now one of the most popular solutions in proteomics. Isobaric labeling-based quantification has many advantages compared to other stable isotope labeling techniques, one of which is the ability to perform high-throughput quantification due to sample multiplexing. Over the years, several different chemical labeling methods have been proposed for isobaric peptide or protein formation. Usually the modification is performed before protein digestion, which requires complete denaturation to overcome errors in quantitation. One of the oldest and cost-efficient methods of isotopically peptide labeling is H3/D3 acetylation and H5/D5 propionylation. This solution is similar to the as isotope-coded protein label (ICPL) technique discussed below, except that the proteins are labeled with H6- or D6-acetic anhydride or H10- or D10-propionic anhydride [17]. In 2002, Zhang and co-workers [18] applied H5/D5 propionylation as a method for phosphopeptide quantification. They used a gentle chemical labeling of N-termini of all peptides with the H5/D5 propionyl group and measurement of abundance ratio of the isotopically labeled/non-labeled pairs using mass spectrometry. The method, although easy to perform and cost-efficient, requires protein denaturation prior to labeling, whereas for complex samples, the presence of the overlapping isotopes clusters may require a high-resolution mass instrument like FT-ICR mass spectrometer (Fourier-Transform Ion Cyclotron Resonance).

## 2. ICPL

**Figure 1:**
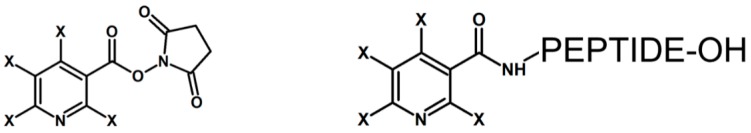
The D4/H4 forms of nicotinyl-*N*-hydroxysuccinimide (Nic-NHS) developed by James et al. [19] and *N*-terminally labeled peptide, which can be applied for quantitation. X-H or D atom.

In 2000, James and co-workers developed a method based on isotopic labeling of all free amino groups in the protein called isotope-coded protein label (ICPL) [19]. The D4 or H4 forms of nicotinyl-*N*-hydroxysuccinimide (Nic-NHS) were used for *N*-terminal nicotinylation of peptides for their quantitative analysis, resulting in a mass difference of 4 Da per label (Figure 1). Lottspeich and co-workers developed H4 or D4-labeled *N*-nicotinoyloxy-succinimide to label free amino groups in peptides for their relative quantitation [20]. The ICPL reagent is now marketed by Bruker Daltonics (Bremen, Germany). In December 2008, Bruker introduced a 4-plex version of this reagent, the Serva ICPL 4plex Kit. This means that there are actually 4 closely related labels: (1) all natural isotopes, (2) X = D, shift of 4 Da relative to all natural isotopes version, (3) X = H, 6 × ^13^C, shift of 6 Da relative to natural version, (4) X = D, 6 × ^13^C, shift of 10 Da relative to all natural isotopes.

## 3. ICAT

**Figure 2:**
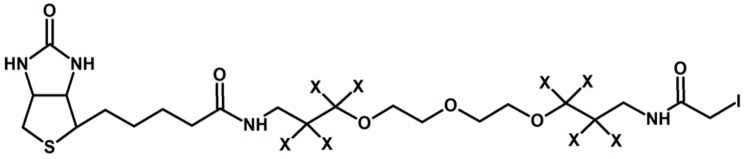
Isotope Coded Affinity Tag proposed by Gygi et al. [21]. X depicts H or D atoms in the molecule.

Gygi and co-workers introduced in 1999 one of the earliest chemical reagents for quantitative proteomics called Isotope Coded Affinity Tag (ICAT) [21]. The proposed solution of labeling involved iodoacetamide as a thiol-reactive group, biotin, no deuterium atoms in the light form and 8 deuterium atoms in a heavy form of the tag (Figure 2). The biotin-avidin pair was used for selective affinity purification of thiopeptides.

A new version of the ICAT reagent, where nine ^13^C atoms were introduced instead of 8 deuteriums and biotin moiety was acid-cleavable, was proposed in 2003 and called cICAT [22,23]. The new form of ICAT reagent eliminates the isotope effect of deuterium during chromatographic separation and removes the potential confusion between a double ICAT label and oxidation, both resulting in a +16 Da mass shift. It was observed that the heavy form is retained stronger than the light form [24]. The use of the ^13^C stable label removed this retention time shift. The known advantage of ICAT reagent is the reduction in sample complexity, because the label specifically targets cysteines, a relatively rare amino acid making up only 1.42% of all amino acids [25]. However, this simultaneously reduces the reliability of the quantification, as the experiment is based on a limited number of peptides per protein. It also makes it impossible to detect changes in the ~10–13% of proteins that do not contain cysteine residues [26,27]. Additional limitation of ICAT is that there are only two label forms available, which could result in multiple experiments if more than two versions need to be compared, and would increase the cost accordingly. The need for comparisons of larger numbers of treatments led to the development of the 2- or 4-plex ICPL, the 4- or 8-plex iTRAQ, and the 2- or 6-plex Tandem Mass Tag (TMT) labeling techniques, which can compare up to four, eight or six samples in a single analysis, respectively. Unlike the previously described labeling techniques, which use the parent ion peak heights or peak areas from the MS spectra, the iTRAQ labels are currently the only tagging technologies commercially available where quantitation is carried out in the MS/MS mode.

## 4. mTRAQ

Mass differential tags for relative and absolute quantification (mTRAQ) are amine-reactive, non-isobaric peptide-labeling reagents introduced by Applied Biosystems, providing the absolute quantification by multiple reaction monitoring (MRM) [28]. The mTRAQ contains a *N*-hydroxysuccinimide ester group enabling primary amine labeling. Specifically, there are two versions of the mTRAQ reagent available: the “heavy” version, which is identical to the iTRAQ 117 tag, and the “light” version that is devoid of any intentional isotopic enrichment. Consequently, labeling of a peptide with the light version adds 140 Da, whereas 144 Da is gained by labeling with the heavy version. This mass difference allows unique MRM transition to be generated for any given peptide labeled with mTRAQ tags. In turn, this implies that, in a sample mixture where peptides originating from different sources are tagged separately with heavy and light labels, the two versions can be monitored independently and distinctly. The mTRAQ comes in three forms, and is not an isobaric tag like iTRAQ [28]. The mTRAQ was designed to be used after the biomarker discovery stage, during the validation stage of a project, as an alternative to having to synthesize deuterated standard peptides for MRM-based quantitation [17]. The precursor-ion mass shifts of these ^13^C and ^15^N-labeled reagents are 0, 4 and 8 Da for arginine and 0, 8 and 16 Da for lysine *C*-terminal tryptic peptides. The mTRAQ technique has already been successfully applied by the Siu laboratory to the quantitation of endogenous levels of a potential cancer biomarker in endometrial tissues [29]. Interestingly, in this study, the expression ratios found by mTRAQ were significantly higher than those found in the iTRAQ-based discovery phase. This provides more evidence of the ‘compression’ of iTRAQ-determined expression ratios.

## 5. iTRAQ and TMTs

**Figure 3:**
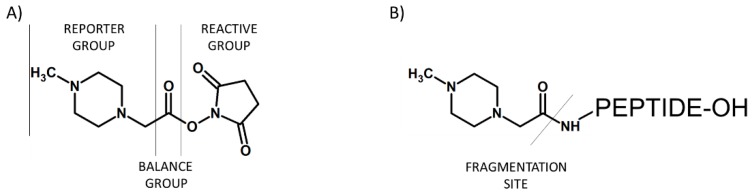
Schematic presentation of iTRAQ structure (**A**) and peptide labeled by iTRAQ (**B**).

In 2004, Ross and co-workers described the isobaric tags for relative and absolute quantification (iTRAQ) [15], which were commercialized by Applied Biosystems. The iTRAQ reagent contains three regions, namely a mass reporter group, a mass balancer group and a protein reactive group *N*-hydroxysuccinimide ester (NHS) that introduces a highly basic group at lysine side chains and at peptide *N*-termini (Figure 3). The first type of iTRAQ reagent, a 4-plex has the variable mass between 114–117 Da within the reporter group.

The modification of 4-plex iTRAQ based on the introduction of larger balancing group resulted in the formation of 8-plex iTRAQ reagent with masses between 113–121 Da in the reporter group. In a MS scan mode, the iTRAQ labeled peptides are characterized by a single peak due to the isobaric masses. The signal intensity summing from all forms of a given labeled peptide in both MS and MS/MS modes increases the sensitivity of detection of analyzed compounds. Additionally, the presence of a basic group having higher affinity to the H^+^ cation under standard ionization conditions increases ionization efficiency. The MS/MS analysis of iTRAQ labeled peptides causes the release of the reporter group in a form of singly charged ions at *m*/*z* 114–117 (for 4-plex) or *m*/*z* 113, 114, 115, 116, 117, 118, 119 and 121 (for 8-plex). The *m*/*z* 120 iTRAQ 8-plex was omitted to avoid overlapping with the phenylalanine immonium ion (*m*/*z* 120.08). The relative quantitation of peptides in the sample is based on the analysis of intensities of these reporter ions in the MS/MS mode. Based on the presence of such reporter ions, the iTRAQ labeling reagent can be applied in the analysis of four different samples using 4-plex kit, or up to eight samples using the 8-plex kit. Both 4-plex and 8-plex are commonly used in the quantitative proteomic analysis of peptides; however, some differences in identification rates have been observed. In 2010, Pichler and co-workers revealed that 8-plex allowed to identify the lowest number of peptides in comparison to 4-plex labeling using iTRAQ or 6-plex TMT [30]. It was concluded that the bulkier structure of the 8-plex label may restrict peptide identification. This results were questioned in 2012 by Pottiez et al., as they achieved more accurate quantitation using 8-plex iTRAQ over 4-plex iTRAQ without sacrificing protein identification rates [31]. However, the observed differences may result from the different instruments and search algorithms used in both experiments [32].

## 6. TMT

**Figure 4:**
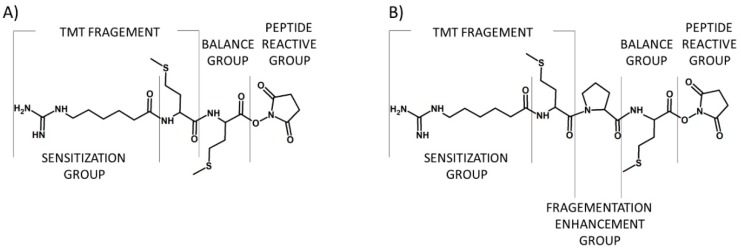
First (**A**) and second (**B**) generation of tandem mass tags developed by Thomson and co-workers [14].

The tandem mass tags (TMTs) are chemical labeling reagents commonly used for mass spectrometry-based quantification and identification of peptides and proteins. The idea of TMTs is based on a similar principle in comparison to iTRAQ, with up to six possible labels. The TMT reagent, proposed by Thompson et al. in 2003, is composed of a mass normalization group (balance group) that balances mass differences from individual reporter ions to ensure the same overall mass of the reagents, a reporter group that provides the abundance of a peptide upon MS/MS in individual samples being mixed, and an amino group reactive functionality making the modification of primary amines possible (Figure 4) [14].

**Figure 5:**
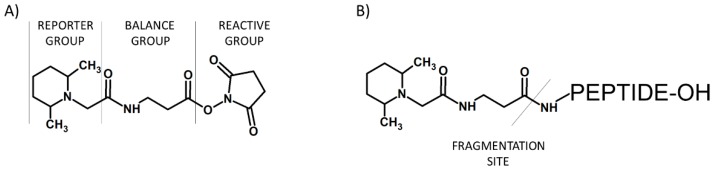
The structure of commercially available TMT (**A**) and the peptide modified by tandem mass tag (**B**) [17].

Several TMT reagents are commercially available as TMTzero, TMT duplex, TMT 6-plex, and TMT 10-plex. They have the same chemical structure but contain different numbers and combinations of ^13^C and ^15^N isotopes in the reporter group (Figure 5). The chemical structure of the TMT tag enables the introduction of five heavy isotopes (^13^C or ^15^N) in the reporter group and five heavy isotopes (^13^C or ^15^N) in the balancer group to provide six isobaric tags. Each of the six tags of TMT 6-plex has a specific reporter ion that appears at *m*/*z* 126, 127, 128, 129, 130, and 131.

TMT 10-plex is an expansion of TMT 6-plex generated by combining current TMT 6-plex reagents with four isotope variants of the tag with 6.32 mDa mass differences between ^15^N and ^13^C isotopes [32]. Even though the mass difference between these reporter ion isotopologues is incredibly small, current generation high resolution and high mass accuracy analyzers can resolve these ions. The numbers of identified peptides and proteins in shotgun proteomics experiments have been compared for the three commercially available isobaric mass tags: iTRAQ 4-plex, TMT 6-plex, and iTRAQ 8-plex [30,32]. Even though the number of identified proteins and peptides was highest with iTRAQ 4-plex, followed by TMT 6-plex, and lowest with iTRAQ 8-plex, the precision at the level of peptide−spectrum matches and protein level dynamic range was similar.

Recently, to increase the multiplexing capacity of quantitative strategies, isobaric labeling and mass difference labeling have been combined [33]. One of the proposed strategy includes triplex labeling with TMT 6-plex, which achieved 18-plex quantitation and with the addition of medium and heavy sets of 6-plex TMT, 54-plex quantitation was demonstrated. Reagents have been produced to permit 2, 4, 6, 8, 10, and 12-plex comparisons. With the use of isotopologues, where the 6.32 mDa mass difference between positional variations of ^13^C and ^15^N substitutions are combined (as are currently used in the 10 and 12-plex reagents), multiplexing up to 18-plex has been suggested as a future possibility [34].

Previously, Sonnet and co-workers devised a system of protein quantification using complement reporter ions (TMTc), which nearly eliminates interference [35]. Additional complement reporter ions are formed as a result of the intact peptide remaining fused to the mass balancing region of the TMT tag. The TMTc ions encode different experimental conditions in the same way the low *m*/*z* reporter ions do, with the added benefit that the TMTc ions’ mass is different for each peptide. The TMTc quantification does not need the additional gas-phase isolation step of the slow MS3 scan; it holds potential to be significantly more sensitive and is compatible with comparatively simple instruments like iontrap Orbitraps, quadrupole Orbitraps, and Q-TOFs.

However, the commercially available reagents for isobaric peptides labeling (TMT and iTRAQ) have some drawbacks, such as high cost in experiments, especially in quantitation for the modified peptides, and inconvenient handling for variable sizes of samples. Recently, Ren and co-workers [36] developed a set of 10-plex isobaric tags (IBT) with high stability and low cost, which is an improved version of 6-plex DiART. IBT 10-plex reagents were applied for dynamic monitoring of the quantitative responses of stimulated phosphoproteome.

## 7. Advantages and Disadvantages

The iTRAQ and other isobaric-tag labeling are performed on digested protein; therefore, every peptide formed during proteolysis should be labeled. The detection of multiple peptides originating from one protein may be achieved providing multiple quantitation per protein. This feature makes possible the identification and quantification of low-abundance proteins in complex samples. The ability of analysing of up to eight samples in parallel suggests that iTRAQ and similar mass balanced labeling tags offer the most promise for quantitative biomarker discovery [37]. However, the disadvantages of the described labeling methods include possible variability in labeling efficiencies, difficulties in protein digestion, and price of the reagents. The protocol of sample preparation involves several steps to achieve reproducible and reliable results. The increasing multiplexicity of labeling reagents also entails a necessity of application of high resolution mass spectrometer to avoid erroneous analysis.

## 8. DiLeu

**Figure 6:**
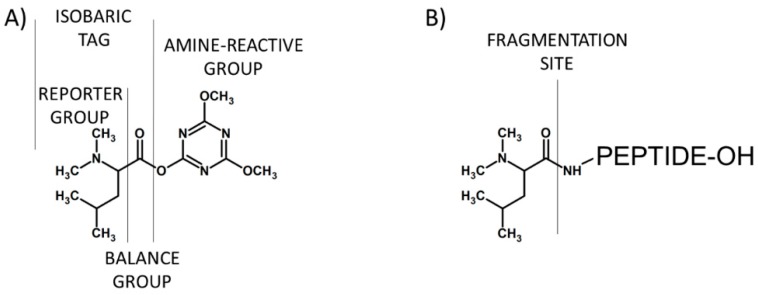
*N*,*N*-dimethyl leucine (DiLeu) tag (**A**) developed by Xiang and co-workers [10] and DiLeu modified peptide (**B**).

The attractive alternative for iTRAQ and TMT based on the *N*,*N*-dimethyl leucine (DiLeu) was proposed in 2010 by Xiang and co-workers (Figure 6) [10]. DiLeu provides relative quantification of peptides and amine-containing metabolites [38]. The amino-reactive group of this reagent targets the *N*-terminus and ε-amino group of the lysine side chain of a peptide. During the MS/MS experiment, this isobaric-labeling reagent produces reporter ions at *m*/*z* 115, 116, 117, and 118. Other characteristic features of DiLeu reagent are modest mass of the tag (mass shift of 145.1 Da), higher intensity of reporter ions compared to iTRAQ and TMT, and enhanced collision-induced MS^n^ fragmentation of labeled peptides at reduced collision energies.

In 2015, inventors of the DiLeu isobaric labeling reagent developed the synthesis of a 12-plex set of DiLeu reagents, which was made possible by exploiting subtle mass differences imparted by mass defects between ^12^C/^13^C, ^14^N/^15^N, and ^1^H/^2^H [39]. This approach enabled a 3-fold increase in multiplexing capacity, keeping the simplicity of synthesis characteristic for the 4-plex set of reagents. However, the small mass difference of ~6 mDa separating the 115, 116, 117, and 118 variants of the 12-plex DiLeu reporters can be baseline-resolved for accurate quantitation at an MS^n^ resolving power of 30 k (at 400 *m*/*z*), which is achievable on the Orbitrap, FTICR, and some QTOF instruments. Acquiring at a resolving power of 60 k baseline resolves isotopic peaks and allows more accurate isotopic interference correction at full multiplexing capacity. Reduced multiplexing configurations allow for highly accurate 9-plex and 7-plex quantitation at resolving powers of 30 k and 15 k, respectively. Additionally, Frost and co-workers [40] presented a cost-effective chemical labeling approach that couples duplex stable isotope dimethyl labeling with 12-plex DiLeu isobaric tags in a combined precursor isotopic labeling and isobaric tagging (cPILOT) strategy. It was found that this approach is compatible with a wide variety of biological samples and permits 24-plex quantification in a single LC-MS/MS experiment.

## 9. DiART

**Figure 7:**
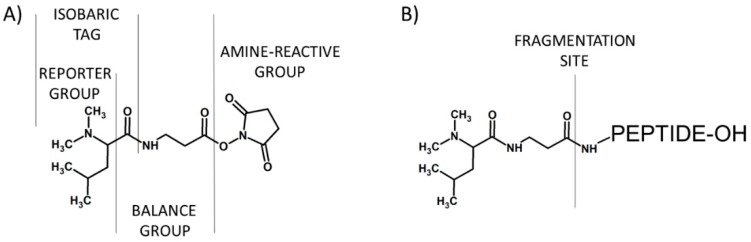
Schematic presentation of Deuterium isobaric Amine Reactive Tag (DiART) [32] (**A**) and DiART modified peptide (**B**).

A good alternative to iTRAQ and TMT for isobaric tagging in quantitative proteomic analysis is Deuterium isobaric Amine Reactive Tag (DiART) [41,42]. DiART reagents consist of an amine-reactive site (NHS ester) for coupling, a balancer, and a reporter (*N*,*N*′-dimethylleucine) with a *m*/*z* range of 114–119 (Figure 7). This labeling reagent uses the same labeling protocol as TMT and iTRAQ.

The application of DiART allows labeling and MS analysis up to six samples. This reagent is characterized by high isotope purity; therefore, the isotopic impurities’ correction characteristic for iTRAQ, TMT, or DiLeu labeling is not required during data analysis of DiART-labeled samples. DiART tags allow generation of high-intensity reporter ions compared to those with iTRAQ. In comparison to iTRAQ or TMT reagents, the reporter ions of DiART may easily undergo fragmentation; therefore, a lower collision energy during MS/MS experiment is required.

### Protein Post-Translational Modifications

**Figure 8:**
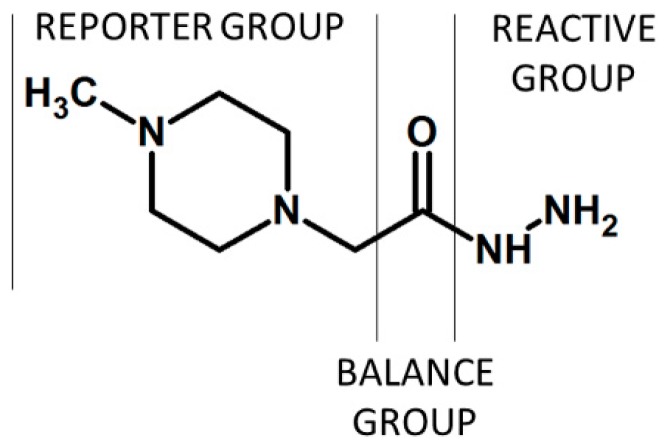
iTRAQ hydrazide (iTRAQH) proposed by Palmese et al. [12].

Co- and post-translational modifications pose a particular challenge to proteome analysis; however, they are frequently transient, substoichiometric, or chemically unstable (or all of the three), and may negatively affect analysis by mass spectrometry, for example by interfering with ionization or fragmentation [43]. Isobaric labeling-based quantification was successfully applied in the analysis of post-translational modifications of some protein, including carbonylated residues in protein. The iTRAQ hydrazide (iTRAQH) was proposed by Palmese et al. as a novel reagent providing the selective labeling of carbonyl groups in proteins and their relative quantification (Figure 8) [12]. The reaction between iTRAQH and carbonylated peptide involves the hydrazine moiety. The iTRAQH reporter ions in the low *m*/*z* region of the MS/MS spectrum provide relative abundance information on the carbonylated proteins in the analyzed samples.

**Figure 9:**
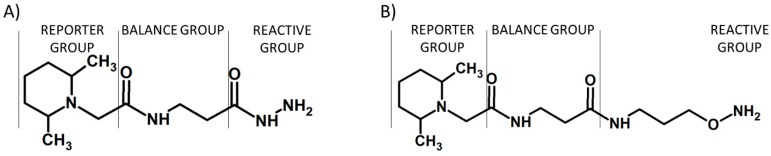
Schematic presentation of the glyco-TMT reagents containing hydrazide (**A**) or aminoxy carbonyl-reactive groups (**B**) [13].

The proposed solution facilitates the analysis of carbonylated peptides by elimination of some sample preparation steps, including enrichment of modified peptides prior to LC-MS/MS analysis. Additional isotopically-labeled carbonyl-reactive tandem mass tags (glyco-TMTs) have been developed and successfully applied for quantification of *N*-linked glycans [13]. These tags are TMT derivatives, containing hydrazide or aminoxy carbonyl-reactive groups (Figure 9).

The proposed glyco-TMT tags can be applied in the quantification of a broad range of biologically important molecules containing carbonyl groups, including oxidized proteins, carbohydrates, and steroids. The aminoxy-functionalized glyco-TMTs are commercially available from Thermo Scientific (Rockford, IL, USA) as aminoxyTMT zero and aminoxyTMT sixplex reagents.

Cysteine, a proteinogenic amino acid containing thiol group, plays a crucial role in protein structure and function. Moreover, the cysteine sulfhydryl groups in proteins are potential sites of reversible oxidative modification because of the unique redox chemistry of this amino acid [11]. It was found that 91% of the proteins contain at least one cysteine residue, which is present in more than 24% of the predicted tryptic peptides [44]. That makes the cysteine-containing peptides an attractive target for proteomic analysis.

In 2012, Tambor et al. [45] reported a novel method of cysteine-containing peptide quantification based on amine-reactive iTRAQ labeling with enrichment of thiol-containing peptides in an approach named cysTRAQ. The proposed methodology allows deeper sampling of the cysteinyl proteome in a manner similar to the ICAT reagents [32].

**Figure 10:**
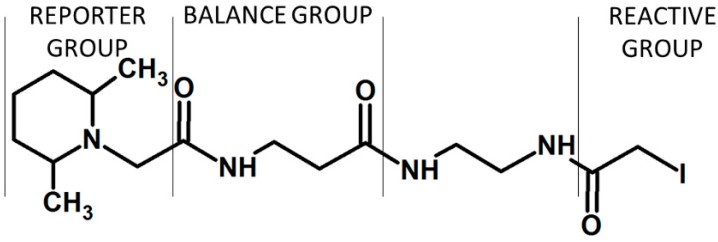
The iodoTMT reagent for cystein-containing peptide labeling and quantification proposed by Pan and co-workers [46].

In 2014, Pan et al. developed a selective reagent for isobaric labeling of cysteine-containing peptides based on TMT, containing the iodoacetyl reactive group (Figure 10) [46]. iodoTMT zero and iodoTMT sixplex are commercially available from Thermo Scientific (Rockford, IL, USA). The proposed isobaric iodoTMT 6-plex reagent within a set has the same nominal mass and consists of a thiol reactive iodoacetyl functional group for covalent and irreversible labeling of cysteine, a balancer, and a reporter group. The iodoTMT sixplex has been commonly used to specifically detect and quantify protein S-nitrosylation [47]. The proposed iodoTMT reagent facilitates the procedure of proteomic analysis of cysteine-containing peptides by reducing the necessity of their enrichment.

The isobaric labeling-based proteomic has many advantages in comparison to other stable isotope labeling techniques, one of which is the ability to perform high-throughput quantification due to sample multiplexing. The ability to combine and analyze several samples within one experiment eliminates the need to compare multiple LC-MS/MS data sets, thereby reducing overall analytical time and run-to-run variation. The isobaric mass tags also offer advantages in the MS/MS mode—there is no interference with peptide fragmentation, as the peptide length distribution profile and amino acid content of the isobarically derivatized peptides are similar to those obtained using other MS-based approaches [48]. In fact, isobaric tags have been reported to improve the efficiency of MS/MS fragmentation and result in increased signal intensities of native peptides in samples of human parotid saliva that, in general, lack the uniform architecture of tryptic cleavage products, e.g., a basic C-terminal amino acid residue [49]. Isobaric labeling-based methods in proteomics have been successfully applied in the analysis of endogenous levels of a potential cancer biomarker in endometrial tissues [29], quantification of human plasma samples [39], and other organisms [17,32]. However, the structure of commercially available isobaric-labeling reagents does not provide a high-proton affinity group and partly introduces hydrophobicity, which may lead to higher ionization efficiency of modified peptides. Additionally, the synthesis of ^13^C, ^15^N and ^18^O isotopically labeled reagents is relatively expensive. Access to deuterated compounds could be significantly more efficient and cost-effective by exchange of hydrogen by deuterium in the target molecule than by de novo synthesis. However, the deuterated compounds are not considered as good internal standards due to the possible chromatographic deuterium effect, which may affect the isotopologues’ co-elution during LC-MS. The synthesis of isotopically labeled tags could be performed using commercially available precursors; however, it frequently involves long synthetic routes and high costs of starting materials. Therefore, labeling by direct hydrogen/deuterium exchange may be faster and a more cost-effective method for preparation of isotopically labeled reagents. Recently, we observed an unusual hydrogen-deuterium exchange (HDX) at the α-C of acetylated *N*-methylglycine (sarcosine) residue in peptides, which occurs in 1% *N*,*N*,*N*-triethylamine (TEA)/D_2_O mixture (pD = 12.3) at room temperature [50]. Additionally, the back exchange (DHX) of introduced deuterons was not observed at neutral pH. The results were confirmed by ESI-MS and NMR data. We also found that such exchange does not occur in the case of *N*-methylalanine residue in model peptides [51]. The method was successfully applied for deuterium labeling of denatonium benzoate (Bitrex) via HDX reaction at the α-carbon situated between carbonyl and the quaternary ammonium group [52]. The proposed strategy is rapid, cost-efficient, and does not require special derivatization reagents or further purification. The LC-MS analysis of denatonium cation isotopologues revealed that the introduced deuterons do not undergo back-exchange under acidic conditions and the co-elution of deuterated and non-deuterated forms was observed. We also developed a method of preparation of deuterium-labeled cyclosporine A standards via H/D exchange of their α-carbon hydrogen atoms occurring in D_2_O under basic conditions [53]. We proved that the preparation of deuterated standards of several different compounds may be obtained by simple H/D exchange, where the cost of the reaction depends only on the price of D_2_O. However, to provide the multiplexity required in the design of isobaric reagents for quantitative proteomics, the introduction of other isotopes is required, which is discussed below.

## 10. Sensitivity Problem

Sensitivity of detection is one of the most important issues in mass spectrometry, which in combination with liquid chromatography systems allows to analyze enormously complex mixtures of peptides. Although this technique has been considered as the most versatile and sensitive for analysis of peptides, there are many problems and limitations in analysis of trace amounts of peptides. This issue results from insufficient ionization efficiency of several peptides, which in turn causes the limited detection of these compounds. Moreover, the insufficient intensity of fragmentation peaks may result in ambiguous sequencing, which impedes the successful interpretation of obtained data.

The aforementioned techniques based on the formation of isobaric peptides as well as the multiplexion of samples do not solve the problem of analysis of trace amounts of peptides. Although the multiplexion has been very useful and enables analysis of up to 12 samples simultaneously (12-plex), it may result in decrease of sensitivity. According to literature data and protocols of producers, 25–50 µg of tagged protein has usually been used for the iTRAQ experiment. However, there are some losses during sample cleanup; therefore, using 200–500 µg of proteins is recommended [54,55]. Thus, a great effort is made on improving the sensitivity of the analysis.

**Figure 11:**
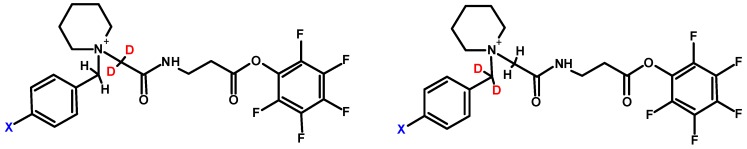
The structures of QAS-iTRAQ 2-plex (X = Br, I) [9].

One of the common practices to increase sensitivity in mass spectrometry is based on the introduction of permanent charge to the molecule of interest, thereby further ionization (i.e., protonation) is not required for the detection. This approach includes formation of quaternary ammonium salts (QAS) [56,57,58] or phosphonium salts (QPS) [59]. The commonly used example of derivatization was coupling of a peptide with *N*,*N*,*N*-trimethylammonium acetyl (TMAA, betaine). Although the introduction of fixed charge resulted in observation of N-terminal fragment principally, the QAS modified peptides did not exhibit signal enhancement [60]. The comparison of signal intensity for derivatized and unmodified compounds showed the higher peaks for unmodified peptides. One of the possible reasons is improvement of the hydrophilic character of peptide due to the presence of fixed charge. Thus, there was a need to change the TMAA for more hydrophobic group, which in turn allowed to overcome this issue. It was also described that the *N*,*N*,*N*-trialkilammonium modified peptides undergo Hofmann elimination [61]. This phenomenon has been regarded as problematic, since it complicates the analysis of obtained data. Moreover, the induction of peptide degradation via intramolecular cyclization was observed. Setner and co-workers solved this problem developing QAS-peptide conjugates based on bicyclic 1-azabicyclo[2.2.2]octane (ABCO) and 1,4-diazabicyklo[2.2.2]octane (DABCO) [62,63]. These structures are cyclic, thereby not susceptible to Hofmann elimination. The sequencing of peptides containing the above-mentioned modifications is facilitated, due to the presence of mainly *a* and *b* ion type series. One of the most interesting features of ABCO and DABCO modified peptides is high tolerance for salt concentration, which means that peptides can be detected even in the presence of 10 mM NaCl. This may be of interest during the analysis of trace amounts of peptides, especially if there are concerns about the loss of the sample during desalting on solid support. Bąchor and co-workers studied the limit of detection for DABCO modified peptides and showed the possibility of detection of 15 attomole sample using nano-LC-ESI-MRM [64]. Moreover, these results were used to design a novel quaternary ammonium-based isobaric tag for relative and absolute quantification of peptides (QAS-iTRAQ 2-plex) (Figure 11) [9].

One form of tag is synthesized using commercially available 4-bromobenzyl[d_2_] bromide, the other is a result of facile H/D exchange in the presence of 1% triethylamine, which results in the introduction of two deuterium atoms in the balance group [65]. This new tag liberates stable benzylic-type cationic reporter upon collisional activation. Thus, this approach introduces an ionization tag, which enables isotopic labeling as well as results in increased ionization efficiency. Although this approach is cost-effective and allows sensitive analysis due to the presence of an ionization enhancer, there are still problems related to limited selectivity because of the active ester reactivity.

**Figure 12:**
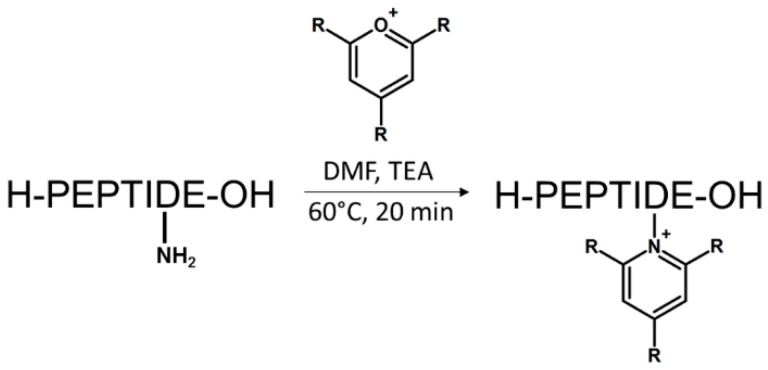
Reaction of peptide with 2,4,6-trisubtituted pyrylium salt (R*-alkyl or aryl).

We recently proposed another class of ionization tags based on 2,4,6-trisubstituted pyridinium scaffold [66]. The reaction relies on a simple conversion of ε-amino groups of lysine to 2,4,6-trisubstituted pyridinium derivative using pyrylium salt; hence, the highly improved sensitivity of peptide detection by mass spectrometry. Pyrylium salts have been known for their reactivity towards sterically unhindered primary amines leading to the formation of pyridinium salt. This chemical property makes them especially selective toward the amine group of the lysine side chain, even in the presence of other nucleophiles such as thiol group. The scheme of reaction is presented in Figure 12.

**Figure 13:**
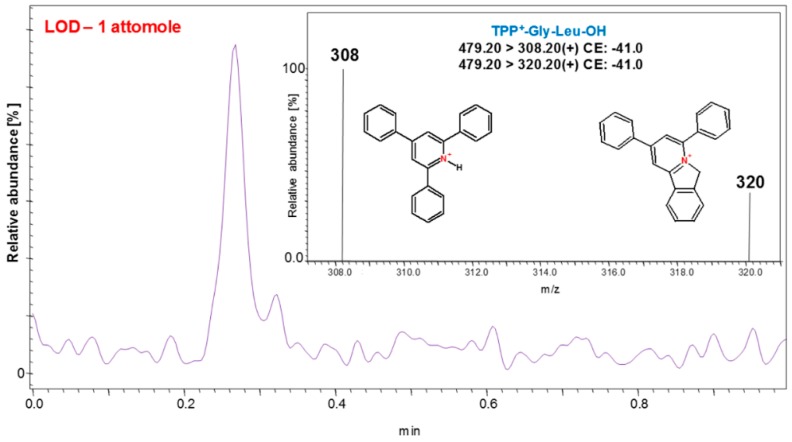
ESI-MS-MRM chromatogram obtained for H-Gly-Leu-OH derivatized with 2,4,6-triphenylpyrylium salt and registered for 1 attomole [66].

It is worth noting that derivatization by pyrylium salt does not require the application of an active ester, e.g., succinimidyl group like in the case of other commonly used derivatizing agents. Thus, there is no risk of a side reaction with the sulfhydryl group of cysteine or the imidazole group of histidine. However, either desalting or lyophilization of the tryptically digested sample is required due to the side reaction with ammonium ions originating from ammonium bicarbonate buffer. Therefore, the use of triethylammonium bicarbonate should be considered. The study on detection limits revealed the possibility of detection of 2,4,6-triphenylpyridinium modified peptide at an attomole level in multiple reaction monitoring mode (Figure 13). The MS/MS fragmentation spectra formed upon collisional activation are characterized mainly by *y* type ions series, due to the presence of permanent charge at *C*-terminus. Thus, the sequencing is facilitated as well as becomes more predictable.

**Figure 14:**
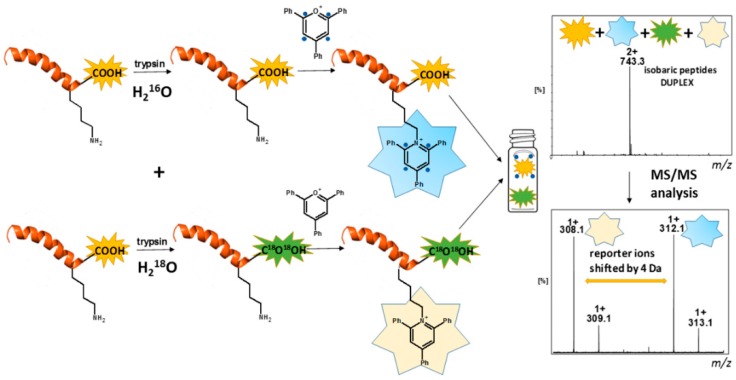
Scheme presenting the formation of a pyridinium-based duplex (the blue circle indicates the ^13^C labeling) [70].

The great advantage of pyrylium salts’ application has been their commercial availability as well as cost-effectiveness. Furthermore, the compounds can be easily synthesized with a good yield by a one-pot cyclization of two equivalents of benzalacetophenone and one equivalent of benzaldehyde [67,68,69]. This fact prompted Waliczek and co-workers to prepare isotopically labeled 2,4,6-triphenylpyrylium (tetra-2,3,5,6-^13^C_4_) and then apply it to the formation of isobaric peptides (2-plex) [70]. This novel approach (Figure 14) exploited the combination of ^16^O/^18^O labeling, commonly used in proteomics and formation of a pyridinium-based ionization enhancer [71,72].

The enzymatic tryptic digestion and simultaneous ^18^O_2_-labeling has become a useful strategy in comparative proteomics and allows for quantitative comparison of two samples during a single LC-MS run. This technique has been featured by easy adaptation to clinical samples, cost-effectiveness, and simplicity of performance. Despite many advantages, there are drawbacks, including peaks overlapping and back-exchange [73]. The first one occurs particularly in multiple charged peptides due to decrease of *m*/*z* difference between the labeled and unlabeled peptide. This may overcome the problem of partial overlapping of the natural isotope peaks of unlabeled and labeled compounds. The scheme of this approach is presented in Figure 14. The two samples are subjected to enzymatic ^16^O/^18^O exchange (trypsin) of the carboxyl group oxygen atoms in the C-terminal lysine residue, while the second one remains unlabeled. The mass difference between these two forms is 4 Da. Then, the ^18^O_2_-labeled first sample is derivatized by 2,4,6-triphenylpyrylium salt, while the unlabeled sample is treated with 2,4,6-triphenylpyrylium salts containing four ^13^C atoms. Finally, the obtained samples are pooled and analyzed by LC-MRM MS. The previous study revealed the presence of an abundant protonated pyridinium cation in the fragmentation spectrum, thereby playing a role of the balancing group (*m*/*z* 308.14 and 312.15) in this method. Thus, a comparison of these two signal allows relative quantitation. It should be noted here that the ^18^O labeling approach possesses some limitations because of incomplete ^16^O/^18^O exchange in case of certain tryptic peptides. Therefore, in rare cases, the proposed method may not be efficient enough and their exclusion from further analysis should be considered. Moreover, this method enables the simultaneous analysis of only two samples and at the current stage there is no possibility to expand it to e.g., 4-plex or 8-plex. However, it is worth noting that a combination of ^16^O/^18^O labeling with derivatization by pyridinium salt solves some problems regarding this approach. First of all, the relative quantitation of peptides relies on comparison of singly charged and is shifted by 4 Da reporter ions, formed as a result of fragmentation of isobaric peptides. Thus, no risk of peak overlapping occurs. Secondly, the pyridinium modified peptides are no longer recognized by trypsin, which in turn prevents the back-exchange. Thirdly, the presence of ionization tag in peptides increases ionization efficiency and allows the sensitive analysis of tryptic digests.

Summarizing the presented data, there is still a need for multiplexing of samples, thereby enabling the simultaneous analysis of many samples. However, due to the frequently occurring decrease in sensitivity with increased multiplexity, there is also a great need for application of ionization tags. Thus, a design of the modern isobaric tags should in our opinion involve the ionization enhancer, thus improving the sensitivity of analysis.

## 11. Conclusions

The presented manuscript provides an overview of the current role of isobaric markers in MS-based proteomics. In recent years, state-of-the-art applications in this field have expanded enormously, proving its validity and utility. Currently, the application of labeling strategies dominates in the field of quantitative proteomic and post-translational modifications’ profiling. Due to its success, chemical isobaric labeling methods play a crucial role in proteomics and, supported by recent advances in mass spectrometric instrumentation and bioinformatics tools, may help solve several problems related to the early diagnosis, prognosis, and monitoring of some diseases. It can be concluded that there is not one perfect and universal method for protein quantification; however, several usable methods have been developed. Additionally, the continuous development of mass spectrometers and new multiplex labeling techniques may significantly improve quantitative proteomic analysis. Although the possibilities of wide use of isobaric tracers in quantitative studies of proteins are beyond doubt, their application in screening medical tests is limited due to the high costs of isobaric reagents and too small increase in the sensitivity of measurements. Therefore, new methods of inexpensive labeling of peptide biomarkers are being intensively sought, which will include not only an isobaric labeling function, but also ionization markers, thus significantly reducing the amount of biological material needed for quantitative analysis. It can be expected that in the future the new isobaric markers will be developed with elevated usable features, including the lowest possible price for production of a set of isobaric reagents; high stability of the reagent allowing its long-term storage at room temperature; high reactivity and specificity in reacting with a specific chemical group of biomarkers; ability to smoothly release certain reporter ions from the labeled biomarker during CID, which will provide reliable quantitative results; high multiplexicity of markers, which will ensure the possibility of employing of a large variety and complex distribution of heavy atoms in the reporter and balancing groups; and high sensitivity of the analysis, by using stable ionization markers in the ionizing marker reporter group, such as cyclic quaternary ammonium salts.
